# Stability of individual differences in executive functions in kindergarten children – a microgenetic study

**DOI:** 10.1007/s10339-025-01283-8

**Published:** 2025-06-07

**Authors:** Eva Michel, Julia Gießübel, Anja Grimm, Leonie Wild

**Affiliations:** Department of Psychology IV, University of Wuerzburg, Röntgenring 10, 97070 Würzburg, Germany

**Keywords:** Executive functions, Updating, Inhibition, Shifting, Stability

## Abstract

Executive functions (EF) are higher cognitive processes which are involved in new, complex tasks. EF are often subdivided into three components: updating of working memory representations, shifting between tasks or task rules, and inhibiting predominant reactions or interfering stimuli. Individual differences in EF are often used to predict academic performance. Although the temporal stability of a construct is a necessary condition for its use as a predictor, the stability of EF in children remains unclear. The present study aims to investigate the short-term stability of individual EF performance in N = 57 kindergarten children. They were tested eight times every 2–3 days with an n-back task to measure updating, a colour/shape sorting task to measure shifting, and a go/no-go task to measure inhibition. Four-week stabilities were high for inhibition and low to moderate for updating and shifting. In latent state-trait analyses, half of the variance in inhibition but very small amounts of variance in updating and shifting variance were explained by trait. Moderate to high amounts of variance in all three tasks were explained by state. The results are discussed in terms of the usefulness of the tasks for measuring stable EF in kindergarten age and for predicting later performance.

## Introduction

Executive functions (EF) are higher cognitive processes associated with prefrontal cortex structures, that are involved in new, complex tasks, such as planning and error monitoring. EF undergo a protracted development, with an important period of development during the preschool years (Garon et al. 2008). In adults, they are often subdivided into three separable but interrelated components: updating, shifting and inhibition (Miyake et al. 2000). Updating refers to the continuous actualization of working memory representations, shifting to the ability to flexibly switch between tasks or task rules, and inhibition to the ability not to attend to interfering stimuli or to inhibit predominant reactions. In children, the subdivision of EF is less clear (e.g., Michel and Bimmüller 2023). Differentiation increases during the preschool and kindergarten years, possibly due to cortical maturation and increasing cognitive demands. However, studies with kindergarten children usually refer to these three core components.

Children´s performance on EF tasks is often used to predict individual differences in development across a wide range of domains, including (pre)school achievement (e.g., Blair and Diamond 2008; Brock et al. 2009; Bull et al. 2008; Michel et al. 2020, 2024; Vandenbroucke et al. 2017), intelligence (Brydges et al. 2012; Roebers et al. 2014), or emotional development (Carlson and Wang 2007; Hughes and Ensor 2011). However, most of these studies do not address EF stability, i.e., the extent to which performance differs between different measures. This neglects the fact that results from, for example, regression analyses are not meaningful if the rank positions of individual performance vary greatly between different measures (Wentura and Pospeschill 2005).

Thus, the present study investigates the stability of interindividual performance differences on common EF tasks for kindergarten children. In the following sections, we review the existing literature on EF stability in children and outline the research questions.

### Interindividual stability of EF in children

In general, the interindividual stability of a construct is assessed using test–retest correlations across measurement points, to quantify rank order stability within groups of individuals (Terracciano et al. 2010). If individuals´ abilities change differently over time, performance is unstable (Friedman et al. 2016), and past performance is not indicative of future performance. To separate the *stability* of a construct (i.e., the amount of variance determined by the trait) from the *reliability* of the measure (i.e., the amount of variance determined by both state and trait variance), latent state-trait analyses are required (e.g., Hagemann and Meyerhoff 2008). “State” factors in children could be, for example, their current mood, the experimenter´s liking, or the quality of their sleep last night. However, the studies described in the following sections mainly refer to stability scores in terms of retest-reliability, i.e., trait and state are not distinguished.

Short-term EF stability is rarely investigated. For longer time periods, studies using EF composite scores suggest moderate to high stability in early childhood. For example, EF composite scores were moderately stable from 24 to 39 months (Carlson et al. 2004). Consistent with this, an EF latent variable at 4.5 years strongly predicted EF at 5 years, indicating longitudinal invariance (Weiland et al. 2014). The same was true for a large sample of children tested on six EF tasks at 3, 4 and 5 years of age (Willoughby et al. 2012). The results showed highly stable rank positions.

However, contextual factors may destabilise EF development: A recent study (Helm et al. 2020) examined EF stability across the transition to school, with assessments at ages 4 and 6. Approximately 13% of the variance in the EF composite score at age 6 was explained by performance at age 4. For 5- to 7-year- olds, Roebers et al. (2011) examined EF stability in a two-year longitudinal study with different cohorts, facing or not facing the transition to school. Half of the children attended a regular play-based kindergarten and a regular school, while the other half attended mixed-grade school classes, an innovative school evaluation project in Switzerland that allows for a flexible and gradual transition to formal learning between the ages of 4 and 8. The EF measures showed considerable one-year stabilities, with performance at t1 explaining about 50% of the performance one year later. Interestingly, the exception was the group of children facing transition to school: Inhibition and updating performance showed low stability coefficients, indicating large changes in rank position.

Despite such contextual influences, individual differences in EF development and EF stability appear to be mainly due to genetic factors: In a large 7-year longitudinal twin study (Polderman et al. 2007) with reaction time data from multiple EF tasks collected at 5 and 12 years of age, longitudinal correlations were substantial, and genetic factors partially mediated stability over time. In line with this, Friedman et al. (2016) showed that EF is quite stable over time due to high genetic correlations. However, the participants were 17–23 years old, so these findings cannot be easily generalised to kindergarten age.

Taken together, EF composite scores appear to be moderately stable over longer periods of childhood. However, when the three core EF components are considered separately, the results are more heterogenous, as shown in the following sections.

### Updating stability

Studies reporting updating stability scores tend to use complex span tasks. This type of task aims to measure the simultaneous storage and processing of information by combining a recall task with a secondary processing task (Schmiedek et al. 2009), such as recalling digits in backward order. The one-year stability of initially 5-year-olds in a backwards digit span task was moderate (*r* = 0.45; Usai et al. 2014). Within a stable educational context, one-year stability of 5- to 7-year-olds in a complex span task was moderate in the longitudinal study mentioned above (initially 5-year-olds: *r* = 0.46; initially 6-year-olds: *r* = 0.43; Roebers et al. 2011). In older children (9–11 years), the one-year stabilities of complex span tasks were moderate to high (operation span: *r* = 0.56; reading span: *r* = 0.71; Hitch et al. 2001). In a recent longitudinal study from kindergarten to grade 2, stabilities of complex span tasks were remarkably heterogenous: while the stability of an odd-one-out task was *r* = 0.32, the stability score of a listening span task was *r* = 0.69 (Kyttälä et al. 2019).

In summary, empirical evidence on interindividual differences in children´s updating performance is heterogeneous, and to our knowledge, no study has yet examined short-term interindividual stabilities at the microgenetic level. Thus, it remains unclear whether kindergarten children´s updating performance is sufficiently stable to justify its use as a predictor of later academic performance.

### Inhibition stability

In contrast to updating, empirical evidence on the stability of inhibition in children is more straightforward. Inhibition tasks can be divided into tasks that measure the inhibition of predominant or ongoing responses (i.e., behavioural inhibition), e.g., go/no-go tasks, and tasks that measure interference control (i.e., attentional inhibition), e.g., Stroop or Flanker tasks). For both types of tasks, performance appears to be moderately stable across childhood. Positive associations between earlier and later performance have been shown across a wide range of task types, age bands and time intervals (e.g., Fujisawa et al. 2017; Kloo and Sodian 2017; Miyake and Friedman 2012; Willoughby et al. 2012). In very young children, longitudinal correlations between inhibition skills at 24 months and an EF composite score at 36 and 48 months were moderate (Stroop task: *r* = 0.24 for 36 months; *r* = 0.28 for 48 months. Gift delay task: *r* = 0.19 for 36 months; *r* = 0.16 for 48 months, Fujisawa et al. 2017). In a longitudinal study with multiple inhibition tasks and five measurement points between 30 and 60 months of age, stability was heterogenous, ranging from *r* =  – 0.02 to *r* = 0.54 (controlled for verbal intelligence), likely due to different tasks used at the different measurement points (Kloo and Sodian 2017). Usai et al. (2014) found a moderate, non-significant 1-year stability for response inhibition in children initially aged 5 years (*r* = 0.32). However, these children faced a transition to school between the two measurement points, which could be a source of destabilisation (Roebers et al. 2011).

Even over very long periods of childhood, inhibition performance remained substantially stable: Polderman et al. (2007) examined selective attention performance longitudinally in a twin study and found moderate stabilities between the ages of 5 and 12 (monozygotic twins: *r* = 0.32, dizygotic twins: *r* = 0.22). These findings were supported by Miyake and Friedman (2012): Interindividual differences in self-regulation in 14- to 36 month olds predicted differences in common EF at the age of 17. In preschoolers (4- to 5 years), delay-of gratification performance even predicted efficiency in a go/no-go task 10 years later (Eigsti et al. 2006). Given the substantial long-term stabilities, it can be assumed that short-term stabilities should be moderate to high, but this has yet to be tested empirically.

### Shifting stability

Findings on shifting stability are heterogeneous, ranging from low to high stability scores, likely due to the wide range of time periods, tasks and age ranges used across studies. Shifting is typically measured with tasks that involve changing rules (e.g., trail-making tasks with alternating colours; van der Ven et al. 2013) or dimensions (e.g., sorting tasks that require children to attend to shape, colour, or size; Willoughby et al. 2017). A very common task type is the Dimensional Card Sorting Task (DCCS). In this task, cards that vary in two dimensions must first be sorted according to one dimension and then, after a rule switch, according to the other dimension (Kloo et al. 2010). In the DCCS task, performance between the ages of 5 (kindergarten) and 6 years (primary school) showed moderate stabilities (*r* = 0.36; Usai et al. 2014). In a recent 2-year longitudinal study of 4- to 6 year-o lds, the stability of the DCCs task was low (*r* = 0.21; Helm et al. 2020). In contrast, Roebers et al. (2011) found higher 1-year stability scores for a simple rule-switching task in 5- to 6-year-olds (initial 5-year-olds: *r* = 0.49, initial 6-year-olds: *r* = 0.57).

In summary, several studies report EF stability coefficients over longer periods of time, although this is usually not the main focus of the research. Short-term fluctuations in children´s ranks have rarely or never been investigated. Moreover, stability coefficients based on correlational analyses cannot answer the question of how much of the observed variance is due to temporally stable individual differences and how much is due to situational effects. Latent-state-trait analyses are needed to answer these questions.

### The present study

The present study aims to shed light on the question of interindividual short-time stabilities of EF performance in children in their last year of kindergarten. EF performance at this age is thought to be predictive of later academic performance. However, the use of EF performance as a predictor is only reasonable if it is- at least – stable over short periods of time. We therefore aim to answer the question of whether individual differences in EF performance are significantly stable within a few days or weeks. Based on the existing research presented above, we hypothesise that inhibition short-term (2–3 days) and 4-week interindividual stability is significant. Hypotheses about shifting and updating are not possible due to the paucity and heterogeneity of results.

In a next step, we will examine whether significant stability scores are indeed indicative of stable underlying constructs, by decomposing the test scores with latent state-trait analyses. More variance in task performance should be explained by trait, i.e., by temporally stable interindividual differences, as opposed to occasion specificity (state).

To measure EF, three well-established tasks were chosen that are widely used with 5- to 6 -year-old children. Updating was measured using an n-back task in which children have to decide whether a stimulus matches a stimulus presented earlier in a sequence (Schmiedek et al. 2009). In contrast to complex span tasks, n-back tasks do not have a fixed end, so they measure the continuous and flexible updating of working memory representations. Performance in complex span tasks and n-back tasks appears to be based on different cognitive processes (Jaeggi et al. 2010; Scharinger et al. 2017).

For inhibition, a go/no-go task was used, because the stability of behavioural inhibition seems to be particularly relevant in the preschool context. Behavioural inhibition is an important aspect of self-regulated classroom behaviour after the transition to school. Due to time constraints, it was not possible to include an additional task measuring interference control. Finally, a computerised version of the DCCS task was chosen to measure shifting, because it is easy to administer and can be used across a wide age range in kindergarten (Zelazo 2006). Although it has been argued that young (3- to 4 year old) children have difficulties with this type of task due to selective attention difficulties (Brooks et al. 2003), it can be assumed that the task measures cognitive flexibility in older kindergarten children (e.g., Moriguchi and Hiraki 2009).

All tasks were computerised, to assess not only accuracy but also reaction time.

In addition to the EF measures, socio-economic status (SES) and age of the children were included as control variables. SES might influence stabilities via associations with supportive parenting behaviour, which has been shown to be positively associated with intraindividual stability of EF performance in 4- to 6-year-olds (Helm et al. 2020). Children´s age should also influence EF performance stabilities, as older children´s cognitive performance fluctuates less than that of younger children (e.g., Dirk and Schmiedek 2016; Galeano Weber et al. 2018; Janvier and Testu 2007).

## Method

### Overview

The sample was recruited from German kindergartens. The children were tested eight times (twice a week, with a time span of 2–3 days between the test sessions) with an n-back task, a colour/shape sorting task, and a go/no-go task. The study followed the principles of the Declaration of Helsinki, and was conducted with the formal approval of the local Ethics Committee of the Institute of Psychology (GZEK 2024-18).

### Sample

The final sample consisted of 57 children (32 boys), with a mean age of 67.07 months (*SD* = 5.64; range = 57–84 months). Socio-economic status was heterogeneous. Analysis with G*Power (Faul et al. 2009) indicated that *n* = 46 would be required to detect medium effects (*f*^*2*^ = 0.30; two-tailed; power = 0.95) in the multiple regression analyses with three predictors. However, due to an error by one experimenter (she incorrectly instructed the children to respond to the no-go stimuli and not to go stimuli), data were only available for 33 children for the go/no-go task. A post-hoc power analysis for *n* = 33 revealed a power of 0.86 (*f*^*2*^ = 0.30; two-tailed) for the regression analyses. For the ANOVAs (repeated measures, within factor; two measures; *f*^*2*^ = 0.30; power = 0.95;), an a priori power analysis indicated a required sample size of *n* = 39, and post-hoc analysis with *n* = 33 indicated a power of 0.92.

Inclusion criteria were being in the last year of kindergarten and having sufficient fluent German language skills, as assessed by kindergarten teachers. Exclusion criteria were intellectual disabilities or language impairment, as assessed by the kindergarten teachers. No child refused to participate or dropped out during the study. All parents gave informed consent, and all children gave assent.

### Measures

*Go/no-go task.* Inhibition of predominant reactions was operationalised with a Pokémon go/no-go task (Durston et al. 2002). Children were instructed to press a button as quickly as possible in response to a visually presented monster, but not to press the button when a rare non-target (a particular monster) appeared. Items were presented in a fixed order (14 no-go trials, 43 go trials). The no-go trials were presented after one, three, or five go trials. The stimulus duration was 500 ms and the interstimulus interval was 3500 ms. The task was programmed in Inquisit 6.6.1, the script was modified based on the original script (Millisecond Software 2022a). Dependent variables were the number of correct no-go reactions and the mean reaction time of the correct go reactions.

*N-back task*. Updating was tested with an n-back task. Children saw a treasure map on which gold coins were presented (stimulus presentation: 500 ms, interstimulus interval: 1500 ms). In the 0-back trials, children had to press a button when the gold coin appeared at a particular position. In the 1-back trials, they had to press the button when the gold coin appeared twice at the same position, and in the 2-back trials, the button had to be pressed when the gold coin appeared at the position of the penultimate coin. There were 16 0-back trials (4 targets), 32 1-back and 32 2-back trials (8 targets). As the working memory load of the 0-back and the 1-back task was minimal, the dependent variables were the number of hits in the 2-back condition and the mean reaction time of these hits.

*Color/shape shifting task.* Shifting was tested using a task adapted from Zelazo (2006). The task was programmed in Inquisit 6.6.1; the script was modified based on the original script (Millisecond Software 2022b). In the first part of the task (“colour game”), children are asked to sort cards with red rabbits and blue boats into the corresponding baskets. Red rabbits should be sorted into the red boat basket, whereas blue boats should be sorted into the blue rabbit basket. In the second part of the task (“shape game”), red rabbits had to be sorted into the basket of the blue rabbit, whereas blue boats should had to be sorted into the basket of the red boat. There were 12 colour trials followed by 12 shape trials (after the rule shift). The dependent variables were the number of correct reactions in the shape trials and the reaction time of the correct reactions in the shape trials.

*Socioeconomic status*. Socioeconomic status was assessed using a questionnaire on the current employment status of both parents. The social position of the parents’ occupations (highest current occupational status) was coded using the International Standard Classification of Occupations (ISCO88; Christoph 2005), with a scale ranging from 20.00 (unskilled labour) to 186.80 (judges). The dependent variable was the sum of the father´s and mother´s scores. If the current occupation of only one parent was recorded in the questionnaire, that score was included.

### Procedure

The tasks were administered individually to each child in a quiet room in the child’s kindergarten, each test session lasted approximately 15 min. Trained psychology undergraduates administered the computerised tasks (on notebook computers with touch screens). All children received a small gift (a small board game).

## Results

For the reaction time measures, outlier analyses were performed at the individual level before the data were aggregated. The Tukey criterion was used. i.e., outliers were defined as those points that fall below Q1–3*IQR or above Q3 + 3*IQR. For the reaction time of the hits in the 2-back trials, 5.4% of the data points were eliminated, for the reaction time of the correct go reactions 0.4%, and for the reaction time of the correct reactions in the shape trials, 2.2%.

Due to the missing values in the go/no-go task, a MCAR test (Little 1988) was used to analyse whether the missing values were completely at random. The results indicated that the hypotheses that the missing values were not random could be rejected (*Chi*^*2*^ (4383) = 0.000; *p* = 1.000).

### Descriptives

**Fig. 1:**
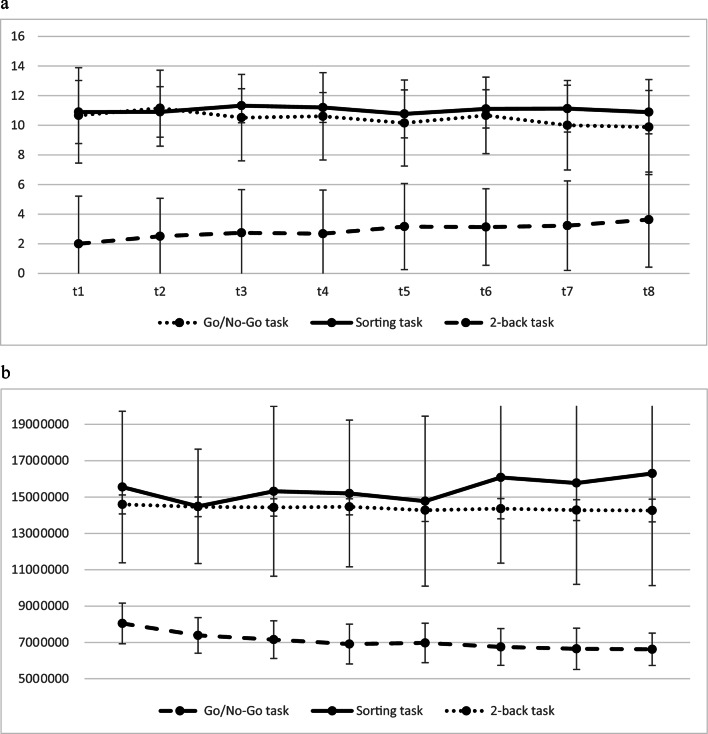
**a** Descriptive statistics (means, standard deviations) of the number of correct reactions for the three EF tasks across the eight measurement points. Go/no-go task: correct no-go reactions. Maximum = 14. Sorting task: Maximum = 12. 2-back task: Maximum = 8. **b** Descriptive statistics (means, standard deviations) of the reaction time variables (msec) for the three EF tasks across the eight measurement points. Go/no-go task: Reaction time of correct go trials. Sorting task: Reaction time of correct “shape” trials. 2-back task: Reaction time of correct 2-back trials

The means and standard deviations for the eight measurement points are shown in Fig. 1. Figure 1a shows the accuracy variables of the three tasks, Fig. 1b the reaction time variables. Task difficulty (i.e., percentage of correct trials) of the 2-back task (hits) was initially high (t1: 25%, t8: 45%). The task difficulty of the go/no-go task (correct no-go reactions) was moderate (t1: 76%, t8: 71%), and the task difficulty of the colour/shape shifting task was low (t1, t8: 91%).

Exact normal distribution was tested with Shapiro–Wilk tests. The test was significant for the correct no-go reactions, t1: *p* = 0.002, t8: *p* = 0.026, for the correct reactions (“shape” trials), t1; t8: *p* < 0.001 for the mean reaction time of the correct reactions (“shape” trials) t1: *p* = 0.021; t8: *p* < 0.001 and for the hits (2-back condition), t1: *p* < 0.001; t8: *p* = 0.006, as well as for the reaction time of the hits (2-back condition), t1: *p* < 0.001; t8: *p* = 0.001. For regression analyses, violations of the normal distribution are not problematic if the sample is larger than 40 (Backhaus et al. 2023). As the go/no-go subsample was smaller than 40, Q-Q plots were examined, which according to Backhaus et al. (2023) showed no systematic violations of the normal distribution.

### Practice effects

**Fig. 2:**
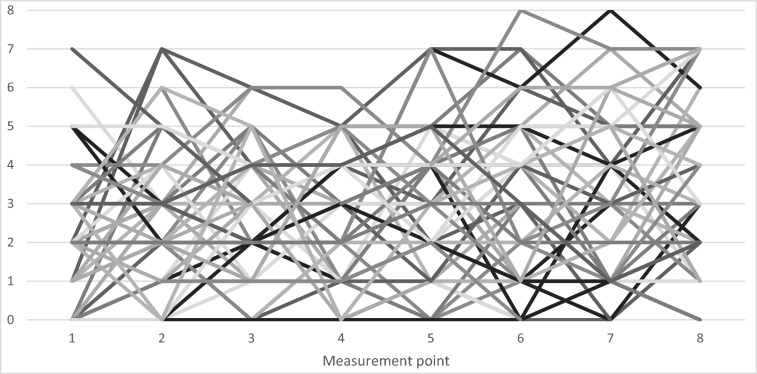
Individual trajectories of the hits in the 2-back task for the eight measurement points

To test for practice effects, repeated measures ANOVAs (t1, t8) were performed, which are very robust to violations of the normal distribution (e.g., Berkovits et al. 2000). For the go/no-go task, the results showed no practice effects for the accuracy of the no-go reactions, but for the mean reaction times (go trials), *F*(1, 31) = 96.81, *p* < 0.001, partial *ƞ*^2^ = 0.76. Children reacted faster at t8 (*M* = 655 ms, *SD* = 80 ms), compared to t1 (*M* = 804 ms; *SD* = 112 ms). For the n-back task, there was a practice effect for the hits (2-back condition), *F*(1, 47) = 26.20, *p* < 0.001, partial *ƞ*^2^ = 0.36, as well as for the reaction times, *F*(1, 52) = 10.01, *p* = 0.003, partial *ƞ*^2^ = 0.16. On average, children had more hits in the 2-back condition at t8 (*M* = 3.63, *SD* = 2.28), compared to t1 (*M* = 2.00, *SD* = 1.79), and reacted faster at t8 (*M* = 1426 ms, *SD* = 62 ms) compared to t1 (*M* = 1458 ms, *SD* = 53 ms). However, the individual trajectories shown in Fig. 2 show significant individual deviations from the mean performance gains. There were no practice effects for the colour/shape sorting task.

### Interindividual stabilities

In order to examine changes in stabilities over time, partial correlations (controlling for the children´s age) between t1 and the other measurement points were analysed for the task variables and the composite scores (see Table 1). SES was not included as a control variable in the correlation analyses because of the overall low interrelations between SES and the EF variables at t1. Composite scores are the z-standardised sum scores for the accuracy variables and the reaction time variables, respectively.

**Table 1:** Partial correlations controlling for age between the first and the other measurement points for the task variables and the composite scores

	Go/no-go: correct no-go reactions	Go/no-go: rt (go trials)	N-back: hits (2-back condition)	N-back: rt (hits, 2-back condition)	Color/shape sorting: correct reactions (“shape” trials)	Color/shape sorting: rt (correct reactions, “shape” trials)	Composite score (rt)	Composite score (correct reactions)
r (t1, t2)	0.62**	0.71**	0.36**	0.28*	0.36*	0.42**	0.75**	0.55**
r (t1, t3)	0.36*	0.57**	0.31*	0.32*	0.56**	0.60**	0.70**	0.08
r (t1, t4)	0.24	0.60**	0.24	0.10	0.53**	0.47**	0.43*	0.59**
r (t1, t5)	0.32	0.58**	0.37*	0.11	0.42*	0.64**	0.54**	0.32
r (t1, t6)	0.46**	0.57**	0.34*	0.11	0.19	0.44**	0.32(*)	0.67**
r (t1, t7)	0.40*	0.34(*)	0.35*	0.40**	– 0.02	0.53**	0.45*	0.24
r (t1, t8)	0.54**	0.66*	0.30*	0.18	– 0.24	0.37**	0.60**	0.40

(*)*p* < 0.10; *p < 0.05; **p < 0.01. Pairwise deletion

*rt* reaction time

Go/no-go stabilities were mostly significant across all time intervals for both accuracy and reaction time variables. Stabilities of the n-back and color/shape shifting task variables were partially significant; effect sizes were small to moderate. The 4-week stabilities (t1, t8) were significant for the go/no-go variables, the accuracy variable of the n-back task and the reaction time variable of the colour/shape shifting task. Interindividual differences on the reaction time variable of the n-back task and the accuracy variable of the colour/shape shorting task were not stable over a 4-week interval. For the composite sores, 4-week stabilities were significant for reaction time, but not for accuracy.

To further investigate whether differences in performance at t8 could be predicted from differences in performance at t1, hierarchical regression analysis was performed with the predictors age and socioeconomic status (step 1) and performance at t1 (step 2). The results are summarised in Table 2.

**Table 2:** Summary of the hierarchical regression analyses to predict t8 performance from t1 performance, age and socioeconomic status

T8 criteria	Significant predictors	β	T	p	Corr R^2^
Go/no-go					
Correct no-go reactions	t1 performance	0.51	3.20	0.003	0.31
Reaction times (go trials)	t1 performance	0.63	4.51	< 0.001	0.39
N-back					
Hits (2-back condition)	Age	0.33	2.36	0.023	
	t1 performance	0.29	2.08	0.044	0.18
Reaction time (hits, 2-back condition)	–				
Shifting					
Correct reactions (“shape” trials)	–				
Reaction time (“shape” trials)	Age	-0.30	-2.20	0.033	
	t1 performance	0.37	2.75	0.009	0.26
Composites score					
Correct reactions	–				
Reaction times	t1 performance	0.56	3.83	< 0.001	0.38

In step 1, age and socioeconomic status were entered. In step 2, t1 performance was entered

For the correct no-go reactions and the mean reaction times of the go trials (t8), as well as for the reaction time composite score, performance at t1 was the only significant predictor. For the hits in the 2-back condition (t8), as well as for the reaction times of the shifting task (t8), age and performance at t1 were significant predictors. No predictor was significant for the reaction times of the hits in the 2-back condition and for shifting accuracy.

### Latent state-trait analyses

Latent state-trait analyses were conducted to determine the proportion of variance explained by consistency (trait) and occasion specificity (state) for the EF components. As the sample size was too small to conduct SEM, we used the simplified parameter estimation from the observed covariances. This has been shown to be superior to SEM analyses in small sample sizes. It provides nearly unbiased estimates of the population parameters in samples as small as *N* = 10 (Hagemann and Meyerhoff 2008).

*Go/no-go task*. First, indicator variables were constructed by splitting the variable “correct no-go reactions” into two test halves (with the first half consisting of the no-go trials 1–7, and the second half consisting of the no-go trials 8–14). Next, the latent trait variance was estimated by computing the arithmetic mean of the covariances of the pairs of indicator variables spanning both measurement points (t1, t8). In addition, the latent state residual variances per occasion (t1, t8) were calculated by subtracting the latent trait variance from the covariance of the respective manifest indicator variables at that measurement point. These indicators were then used to calculate coefficients of consistency (trait variance) and occasion specificity (state variance). The coefficient of consistency for each variable was calculated by dividing the latent trait variance by the variance of the corresponding manifest variable. The coefficient of occasion specificity was calculated by dividing the latent state residual variance per occasion by the variance of the respective manifest variable. The results are presented in Table 3.

**Table 3:** Percentage of variance determined by state and trait for the three tasks and the first and the last measurement point

	% Variance determined by trait	% Variance determined by state
Go/no-go		
t1: first test half/second test half	55/47	28/24
t8: first test half/second test half	40/56	14/20
N-back		
t1: first test half/second test half	< 10/ < 10	83/47
t8: first test half/second test half	< 10/ < 10	18/20
Shifting		
t1: first test half/second test half	< 1/ < 1	56/90
t8: first test half/second test half	< 1/ < 1	23/23

Go/no-go variable: correct no-go reactions. N-back variable: hits in the 2-back condition. Shifting variable: correct reactions in the “shape” trials.

As the reliability coefficient of the manifest variable is the sum of the coefficient of consistency and the coefficient of occasion specificity (Hagemann and Meyerhoff 2008), the reliability score of the first test half at t1 was 0.83, and that of the second test half was 0.71, indicating moderate to high reliability. At t8, the reliability scores were 0.54 (first test half) and 0.76 (second test half).

*Updating*. For the hits in the 2-back condition (split-half subtests), the task was not reliable except from the first test half of t1.

*Shifting*. For the correct reactions in the “shape” trials (split-half subtests), the task was not reliable except from the second test half of t1.

## Discussion

This study examined the stabilities of EF performance in kindergarten children over a 4-week interval and analysed how much variance in EF performance was due to true person and situation effects. The strengths of the study are the large number of measurement points within short time intervals (2–3 days), the use of latent state-trait analysis, and the inclusion of different EF components, which allows stability indices to be compared across tasks.

Short-term (2–3 days) stability of EF task performance was moderate to high overall, but only inhibition performance showed high 4-week stability after controlling for children´s age and SES. Most importantly, about half of the variance in the inhibition task was explained by temporally stable interindividual (trait) differences. In contrast, shifting and updating performance was generally not very stable over time. Within the 4-week test–retest interval, the updating and shifting tasks were unreliable.

The results indicate that kindergarten children´s inhibition performance is highly stable over a 4-week period, consistent with and extending findings demonstrating long-term stabilities (e.g., Fujisawa et al. 2017; Kloo and Sodian 2017; Miyake and Friedman 2012; Willoughby et al. 2012). Furthermore, the go/no-go task appears to be a reliable measure of children´s response inhibition skills, as children’s performance on the go/no-go task reflects individual differences in true (trait) response inhibition performance. The results are consistent with Willoughby et al. (2012), who reported that 25–50% of the variance in EF tasks is due to true ability. Taken together, go/no-go performance in kindergarten may be a reliable predictor of, for example, later academic success in prospective long-term studies.

In contrast, the n-back task in the present study did not measure temporally stable (trait) aspects: The amounts of variance explained by trait were small, and the overall low 4-week test–retest correlations are due to both substantial within-person variability and poor reliability of the measure. The small amount of trait variance may be due to the high difficulty of the task (see Fig. 1a), combined with the large number of measures within a short period of time, potentially maximising the effects of emotional and motivational states. Unsystematic observations during the test situations suggest that the task may have been slightly aversive. Motivation to complete the task may have varied greatly between children and between test sessions. In particular, the last measure is characterised by large measurement error. The reliable and valid measurement of updating performance in kindergarten children with n-back tasks therefore seems difficult. The 2-back task of the present study cannot be used as a reliable predictor to investigate, for example, the predictability of later school performance. In contrast, complex span tasks are often reliable and may be more appropriate for kindergarten age (Usai et al. 2014; Roebers et al. 2011; Hitch et al. 2001). However, it is questionable whether complex span tasks adequately tap executive updating processes to the same extent as n-back tasks, as discussed above.

Considering the rule shifting task in the present study, performance stability was low to moderate, and the task did not measure children´s true (trait) performance. The last measure was completely unreliable. Existing results on the stability of rule shifting in kindergarten children are heterogeneous, so stabilities may be very task specific. The colour/shape task in the present study was very simple, and ceiling effects might have contributed to the low reliability, see Fig. 1a. In addition, the task consisted of only 12 trials (to ensure short test sessions). Overall, this task does not seem suitable for measuring shifting performance in kindergarten children. Another interpretation of the results is that at this age, shifting performance is not very temporally/situationally stable, but is mainly driven by occasional, emotional and/or motivational aspects. Moreover, low stability scores may also reflect different growth rates in the underlying trait, especially in young children. These interpretations are consistent with existing studies that have found low stability scores (e.g., Helm et al. 2020). However, as other research suggests substantial long-term stability (e.g., Roebers et al. 2011; Usai et al. 2014), these assumptions seem premature. Future studies should examine shifting stabilities with different tasks and large samples to enable latent state-trait modelling with SEM.

Overall, two of the three EF tasks are characterised by only small amounts of variance explained by trait and low reliability. This is in line with Willoughby et al. (2012), who state that individual EF tasks are often weak indicators of true ability. They suggest aggregating performance across different EF tasks, which resulted in a stable trait index in 3–5 year old children in their study. This is consistent with Miller-Cotto and Gordon`s (2025) suggestion that EF tasks tap a common executive attention construct and fits well with findings that a three-factor EF structure is often not found at kindergarten age (Michel and Bimmüller 2023). Empirical studies have often found one factor, or two factors with either a combined shifting/updating factor or a combined shifting/inhibition factor. No study found a separate shifting factor in kindergarten age. Stable shifting traits appear to develop later than the other two EF components (Michel and Bimmüller 2023). This is consistent with the measurement problems found in the present study. From a practical point of view, the results of individual EF tasks should therefore be aggregated, e.g., for the longitudinal prediction of performance in academic domains. However, in the present study, only the reaction time composite scores, but not the accuracy scores, were stable over the 4-week interval. This may be partly due to the small sample size and the missing values of the go/no-go task discussed above. Nevertheless, it suggests that response inhibition measures may be more stable than EF composite scores over relatively short time intervals.

From an intervention perspective, the substantial state variance of the EF tasks suggests the potential trainability of EF performance. The effectiveness of EF training in children is controversial, but a recent meta-analysis found benefits for both near and far transfer tasks (Birtwistle et al. 2025). However, the low reliability of some EF tasks suggests a measurement problem in identifying early EF delays: If EF performance is not stable, diagnosing delays based on of a single EF measure is problematic.

A secondary finding of the present study was the absence of systematic practice effects: In the go/no-go task, children responded faster but not more accurately on the last measure compared to the first measure. In contrast, in the 2-back task, children were more accurate and faster on the last measure, compared to the first measure. In the rule shifting task, there were no practice effects. Overall, these findings are consistent with the theoretical assumption that EF tasks should not be easily automated in children, because the shift from controlled to automatic processes would take longer than in adults (e.g., Hughes and Graham 2002). Once automated, the task would no longer measure EF. The individual performance trajectories shown in Fig. 2 emphasise that the go/no-go task did not become automated even after eight test sessions within a relatively short time interval. Thus, the task did indeed measure executive aspects of response inhibition, even after much practice.

### Limitations and future research directions

The sample of the present study was too small to conduct a model-based (SEM) latent-state trait analysis, so the study should be replicated with a larger sample. This would also be important in light of the well-known problem of task impurity in EF measures. EF tasks do not measure single EFs, but a mixture of e.g., inhibition and updating processes, together with other task-specific (e.g., prior knowledge) as well as task-unspecific non-executive processes (e.g., processing speed) (Miller-Cotto and Gordon 2025). An attempt to address this issue in the present study is the use of manifest composite scores, but a latent common EF variable would address this issue much better.

In addition, the tasks contained relatively few trials, which may have affected the test power of the latent variable analyses. However, longer test sessions might have made the repeated tasks (more) aversive for the children. Even with the short tasks used in the present study, completing the same tasks eight times in four weeks may have led to a loss of motivation over time, which could have affected the stability of the rank-order (if children differ in their potential loss of motivation).

Another issue is the potential for task automation when the same task is performed multiple times, potentially reducing the executive demands of the task. However, the large individual fluctuations in performance levels and the unsystematic practice effects suggest that task performance was not automated and that the executive demands of the tasks were not considerably reduced across the measurement points, in line with theoretical assumptions (e.g., Hughes and Graham 2002).

Furthermore, future studies should consider both intra- and interindividual stability: For example, in a previous study, intraindividual day-to-day fluctuations accounted for about half of the variance of interindividual stability in working memory performance (Dirk and Schmiedek 2016). Thus, interindividual stability may be largely determined by intraindividual stability. Furthermore, future research should investigate which factors contribute to EF stability. In the present study, a broad indicator of SES did not account for significant amounts of variance in EF performance. The home literacy environment and parenting behaviour may be more predictive of EF performance in kindergarten children, as well as, for example, daily fluctuations in the kindergarten educational context, or the child´s emotional state.

## Conclusion and practical implications

From a practical point of view, it seems important to increase knowledge about EF stabilities: Do those children who start behind in kindergarten continue to stay behind later? On the one hand, the substantial stability of inhibition performance in the present study underlines the importance of early diagnosis of impaired EF development. On the other hand, the large amounts of variance explained by state in all three EF measures emphasise the changeability of performance and thus the potential of early intervention programmes that focus on accurate and rapid mastery of EF tasks, as well as on motivational and emotional aspects.
